# Supplementary material for: Phenotypic drug susceptibility testing for Mycobacterium tuberculosis variant bovis BCG in 12 hours
Source: Nat Commun. 2025 May 10;16:4366. doi: 10.1038/s41467-025-59736-9 (PMC12065818; doi:10.1038/s41467-025-59736-9)
Supplement: Supplementary file 1 — Supplementary Information [file 41467_2025_59736_MOESM1_ESM.pdf]

Supplementary Information

Phenotypic drug susceptibility testing for *Mycobacterium tuberculosis* variant *bovis* BCG in 12 hours

Buu Minh Tran[1], Jimmy Larsson[1], Anastasia Grip[1], Praneeth Karempudi[1], Johan Elf[1]✉

[1]Department of Cell and Molecular Biology, SciLifeLab, Uppsala University, Sweden

✉E-mail: johan.elf@icm.uu.se

Keywords: antimicrobial susceptibility testing (AST), deep neural network (DNN), drug resistance, microfluidic chip, *M. bovis* BCG, *M. smegmatis*, phenotypic drug susceptibility testing, single-cell imaging, tuberculosis

Chip design and fabrication. To improve the capturing of mycobacteria, we adjusted our chip design (Supplementary Fig. 1) from the Mother Machine chip developed by our group for the phenotypic antibiotic susceptibility test in urinary tract infections[1]. The main feature of the chip is a cell trap region consisting of two parallel rows of 100 microchambers with dimensions of 50×60×1 µm. There are ports of the front channels shared by two rows of microchambers including ports 2.0 (Supplementary Fig. 1a), 3.0, 4.0, 7.0, and 8.0. We used port 2.0 to load the cells. Separate ports for each row of the microchambers including ports 2.1, 3.1, and 4.1 from one side and corresponding to ports 2.2, 3.2, and 4.2 on the other. We used ports 2.1 and 2.2 for media with and without antibiotics. None of the other ports are used in this work.

Each fluidic port has a filter region of gradually decreased filtration size to trap air bubbles and prevent dust or media precipitations from entering the cell trap region. Fluid connects the front and back channels via microchambers. Each microchamber has a dot code on each side to facilitate the image analysis (*e.g.,* cropping and correcting stage shift between the frames) and an array of 300 nm constrictions on the side facing the back channel to trap the cells and allow fluidic flow to the back channel (Fig. 1b). Design of the filter region and dot codes are detailed in the previous work[1].

The mold was produced by the ConScience company (Gothenburg, Sweden). On 1000 µm Si-wafer substrates, mold production occurs in three stages including (1) the SiO2 formation using thermal oxidation, (2) the etching of microchambers and optical alignment through a combination of electron beam lithography, reactive ion, and wet etching, and (3) the generation of flow channels, ports, and filtering regions in a 10-µm-thick SU8 layer using lithography.

The microfluidic chip is a borosilicate cover glass (thickness no. 1.5, 22×40 mm, VWR) bonded with a micromolded silicon elastomer (PDMS). The cover glasses were washed thoroughly under running deionized water, then rinsed in 96% EtOH, and washed again with deionized water before being submerged in 2% (v/v) Helmanex III in MilliQ water inside a Teflon vessel for a cleaning sonication for 45 min. After the sonication, the cover glasses were washed thoroughly and stored in MilliQ water. PDMS base (Sylgard 184; Dow Corning) and the curing agent were mixed thoroughly at 10:1 (w/w), poured on top of the mold, and degassed using a vacuum pump. The curing process was done overnight at 80°C. The cured PDMS was demolded and individual chips were diced. The chip ports were punctured and then surface-cleaned with Scotch Magic Tape (3M), followed by a swift rinse in isopropyl alcohol (Sigma-Aldrich) before drying under clean pressurized air. The micromolded PDMS and cover glass were surface-activated using air plasma at 100 W (~50% power, Henniker Plasma) for 30 seconds, and covalently bonded together. The complete bonded chip was further incubated overnight at 80°C.

Macrofluidic set-up and microfluidic chip wetting before loading cells: We used 15 mL Falcon tubes as macrofluidic reservoirs. The reservoirs are placed on the same level (height) as the microscopy stage. The reservoirs have adapters (Elveflow) connected to the microfluidic chip via tubing (VWR, TYGON VERNAAD04103). There are metal connectors (23-gauge, 14-mm-long tubing bent in the middle at 90°; New England Small Tubing) between the tubing and chip's ports. Before starting the experiments, the microfluidic chips were wetted with growth media in the order of ports as follows: ports 5.1, 5.2, and 6.0 at 800 mbar, port 2.0 at 800 mbar, and then ports 2.1

and 2.2 at 700 mbar (Supplementary Fig. 1). The pressure was regulated using the OB1-Mk3 regulator (Elveflow). After the wetting step, pressure to ports 5.1, 5.2, and 6.0 was stopped. Mycobacteria cells were loaded from port 2.0 at 120 mbar with a counter-pressure from ports 2.1 and 2.2 at 50 mbar. The loading pressure (port 2.0) could be slightly increased when necessary to ensure all microchambers in the trap region were filled with cells. During the experiments, media from ports 2.1 and 2.2 were supplied at 80 to 100 mbar and none of the other ports had pressure applied.

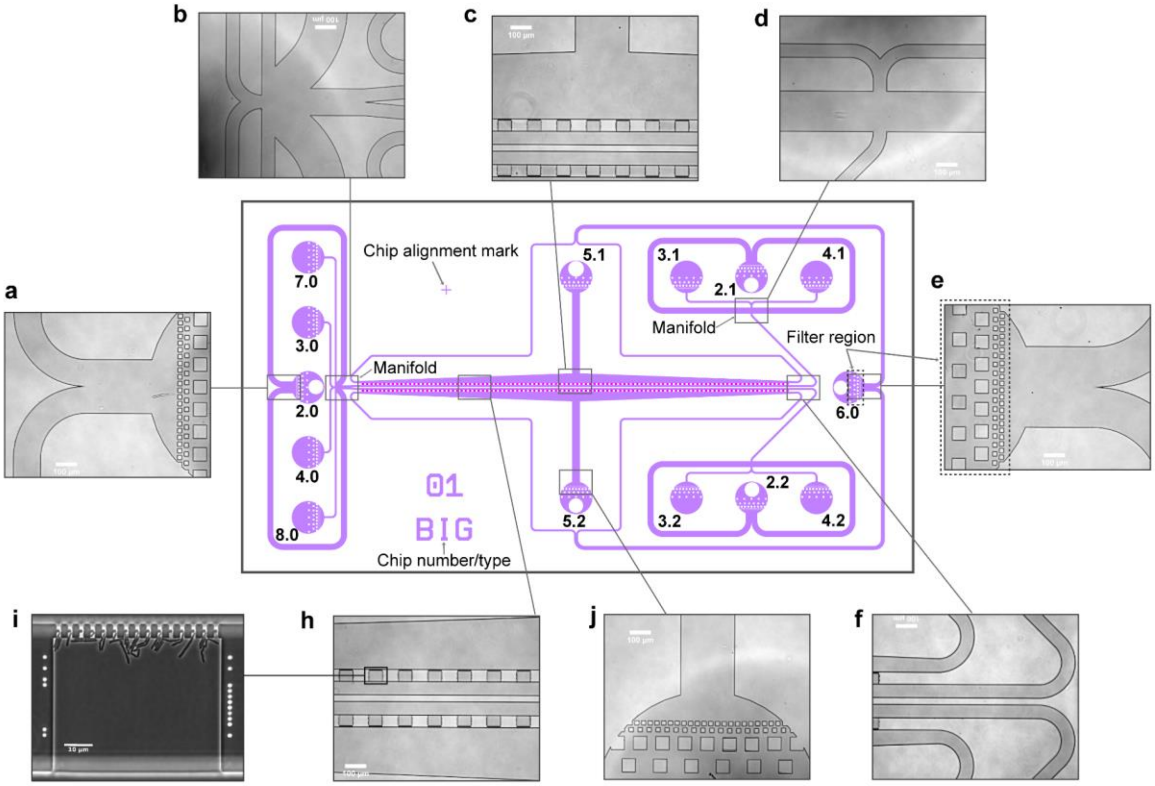

Supplementary Figure 1. Design and features of the microfluidic chip including the drawing design displaying the designated ports, filter region with orientations, chip number, chip type/number, ad manifold positions for multiple modes of operations, and microcopy images of different areas. **a**. One of the ports in the front channel including ports 2.0, 3.0, 4.0, 7.0, and 8.0 - we only used port 2.0 for loading mycobacterial cells. **b**. A region of the loading port manifold. **c**. An area in the middle of the chip shows arrays of microchambers and a part of a back channel leading to port 5.1. A symmetrical structure is designed on the opposite side with a back channel to port 5.2. **d**. A manifold for ports 2.1, 3.1, and 4.1 with a symmetric manifold on the opposite side for ports 2.2, 3.2, and 4.2. We used ports 2.1 and 2.2 to supply media with and without antibiotics. **e**. Port 6.0, together with ports 5.1 and 5.2, is used to maintain back-channel pressure. **f**. Connecting channels of the front and back. **j**. Port 5.2 of one of the back channels. **h**. An area in the middle of the cell trap region. Scale bars are 100 µm. **i**. Phase-contrast image of one of the microchambers in (**h**) with constriction gaps of 300 nm at the end and dot code on each side. Trapped cells are *M. smegmatis* NCTC 8159. The scale bar is 10 µm.

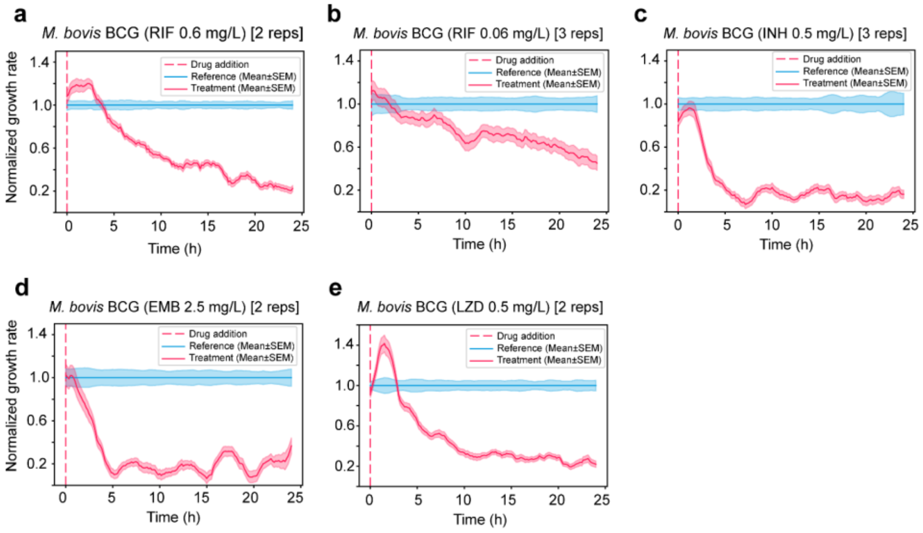

Supplementary Figure 2. pDST assays detecting fast response of *M. bovis* BCG to (**a**) rifampicin (RIF) 0.6 mg/L, (**b**) rifampicin (RIF) 0.06 mg/L, (**c**) isoniazid (INH) 0.5 mg/L, (**d**) ethambutol (EMB) 2.5 mg/L, and (**e**) linezolid (LZD) 0.5 mg/L. Data were pooled from the number of biological replications indicated in each graph.

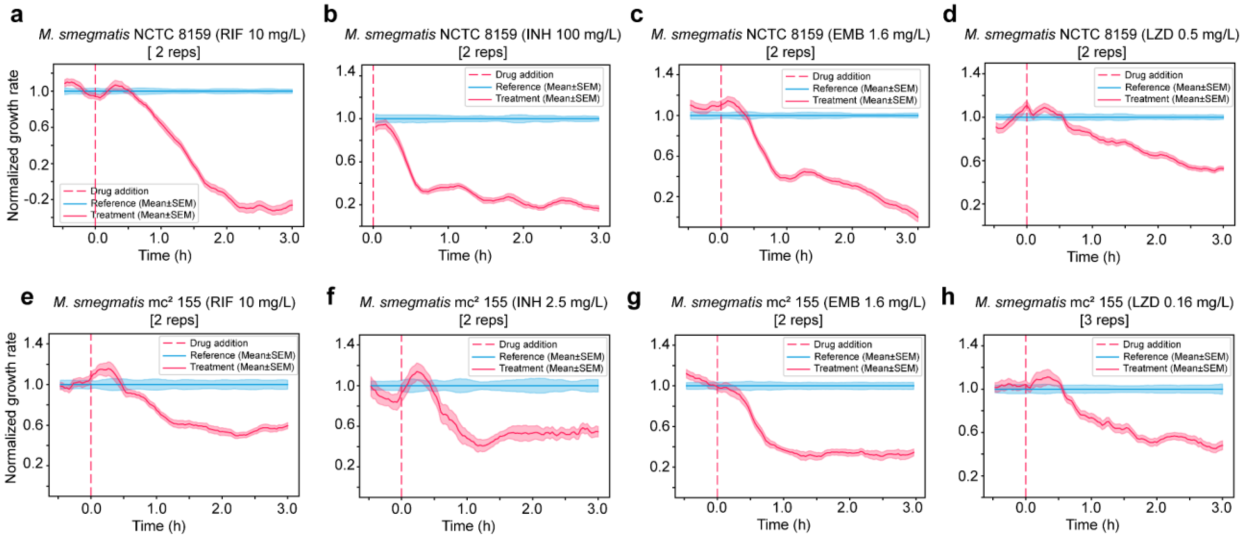

Supplementary Figure 3. pDST assays detecting fast response of *M. smegmatis* NCTC 8159 to (**a**) rifampicin (RIF) 10 mg/L, (**b**) isoniazid (INH) 100 mg/L, (**c**) ethambutol (EMB) 1.6 mg/L, and (**d**) linezolid (LZD) 0.5 mg/L; and *M. smegmatis* mc^2 155 to (**e**) rifampicin (RIF) 10 mg/L, (**f**) isoniazid (INH) 2.5 mg/L, (**g**) ethambutol (EMB) 1.6 mg/L, and (**h**) linezolid (LZD) 0.16 mg/L. Data were pooled from the number of biological replications indicated in each graph.

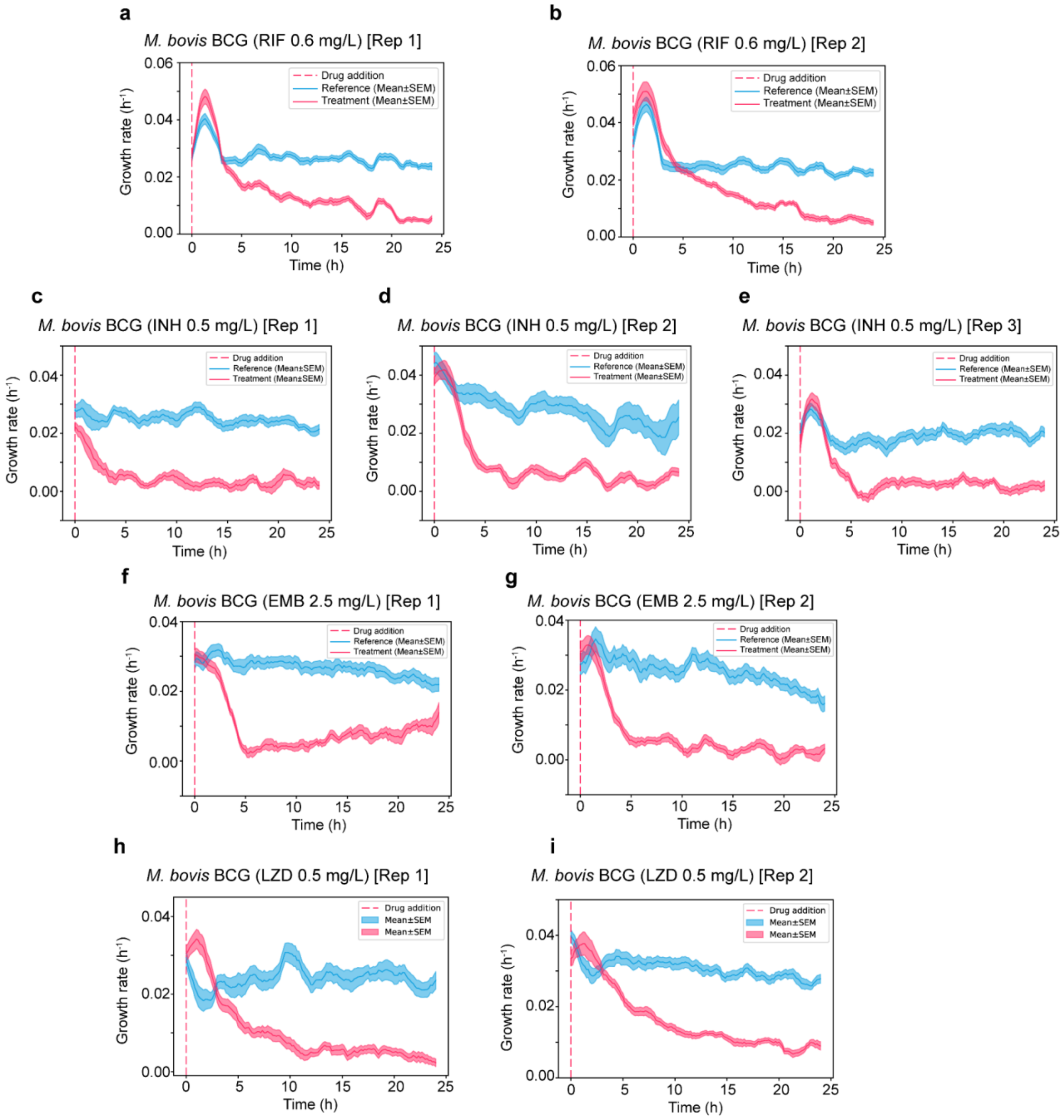

Supplementary Figure 4. The growth rates of *M. bovis* BCG in response to drug treatments were measured across two or three biological replicates from different fast pDST experiments. **a - b**. *M. bovis* BCG in rifampicin (RIF) 0.6 mg/L. **c - e**. *M. bovis* BCG in isoniazid (INH) 0.5 mg/L. **f - g**. *M. bovis* BCG in ethambutol (EMB) 2.5 mg/L. **h - i**. *M. bovis* BCG in linezolid (LZD) 0.5 mg/L.

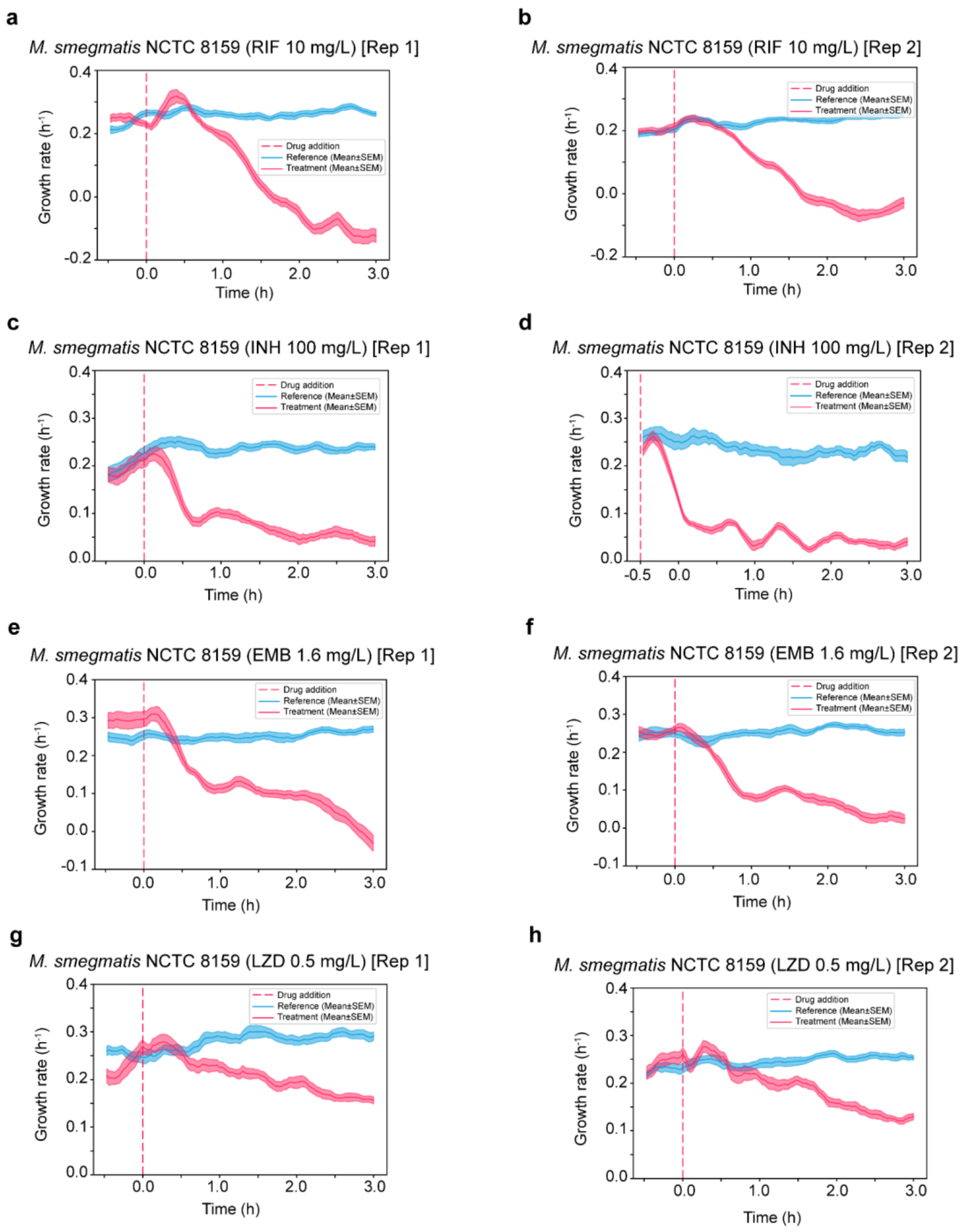

Supplementary Figure 5. The growth rates of *M. smegmatis* NCTC 8159 in response to drug treatments were measured across two biological replicates from different fast pDST experiments. **a - b**. *M. smegmatis* NCTC 8159 in rifampicin (RIF) 10 mg/L. **c - d**. *M. smegmatis* NCTC 8159 in isoniazid (INH) 100 mg/L; In replication 2, the drug was added after 30 min instead of 1 hour like other measurements. **e - f**. *M. smegmatis* NCTC 8159 in ethambutol (EMB) 1.6 mg/L. **g - h**. *M. smegmatis* NCTC 8159 in linezolid (LZD) 0.5 mg/L.

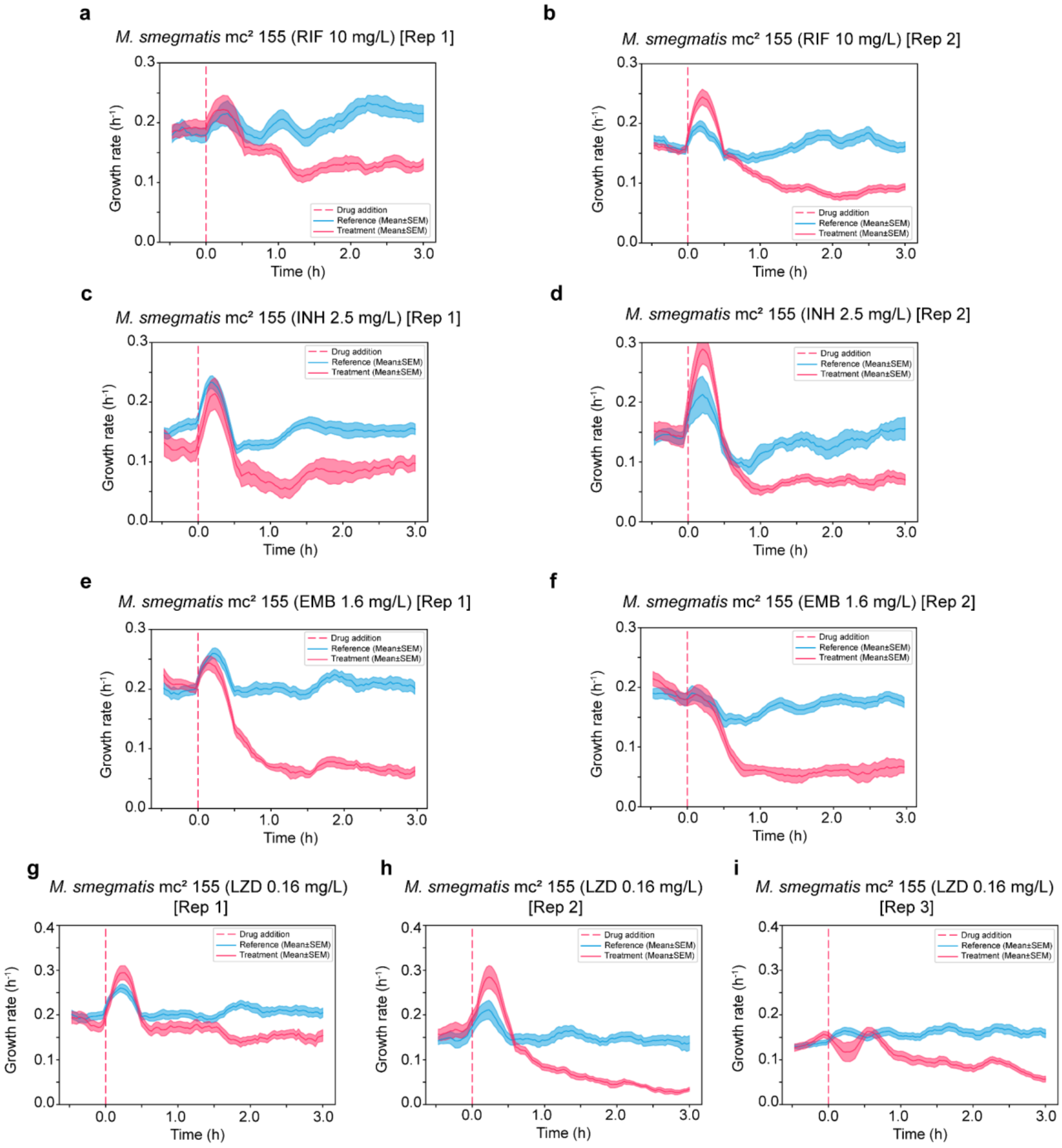

Supplementary Figure 6. The growth rates of *M. smegmatis* mc^2 155 in response to drug treatments were measured across two or three biological replicates from different fast pDST experiments. **a - b**. *M. smegmatis* mc^2 155 in rifampicin (RIF) 10 mg/L. **c - d**. *M. smegmatis* mc^2 155 in isoniazid (INH) 2.5 mg/L. **e - f**. *M. smegmatis* mc^2 155 in ethambutol (EMB) 1.6 mg/L. **g - h**. *M. smegmatis* mc^2 155 in linezolid (LZD) 0.16 mg/L.

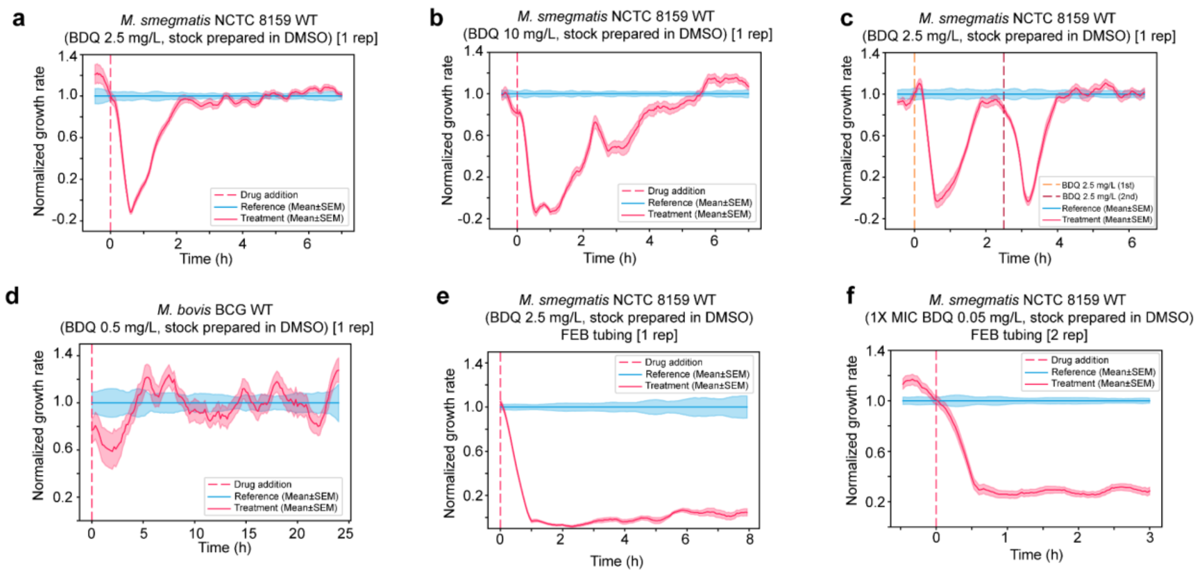

Supplementary Figure 7. Recovery of mycobacteria in BDQ treatments on the microfluidic chip. **a.** *M. smegmatis* NCTC 8159 WT in BDQ 2.5 mg/L (50X MIC), BDQ stock prepared in DMSO; The drug was added at t=0 hour. **b.** *M. smegmatis* NCTC 8159 WT in BDQ 10 mg/L (200X MIC), BDQ stock prepared in DMSO. **c.** *M. smegmatis* NCTC 8159 WT in BDQ 2.5 mg/L (~50X MIC), BDQ stock prepared in DMSO; The drug was added at t=0 hour, and then a freshly made drug was added for the second time at t=2.5. **d.** *M. bovis* BCG WT in BDQ 0.5 mg/L (~4X MIC), BDQ stock prepared in DMSO. **e**. *M. smegmatis* NCTC 8159 WT in BDQ 2.5 mg/L (50X MIC), BDQ stock prepared in DMSO, using chemically inert fluorinated ethylene propylene (FEP) tubing. **f**. *M. smegmatis* NCTC 8159 WT in BDQ 0.05 mg/L (1X MIC), BDQ stock prepared in DMSO, using FEP tubing. The number of biological replications is indicated in each graph.

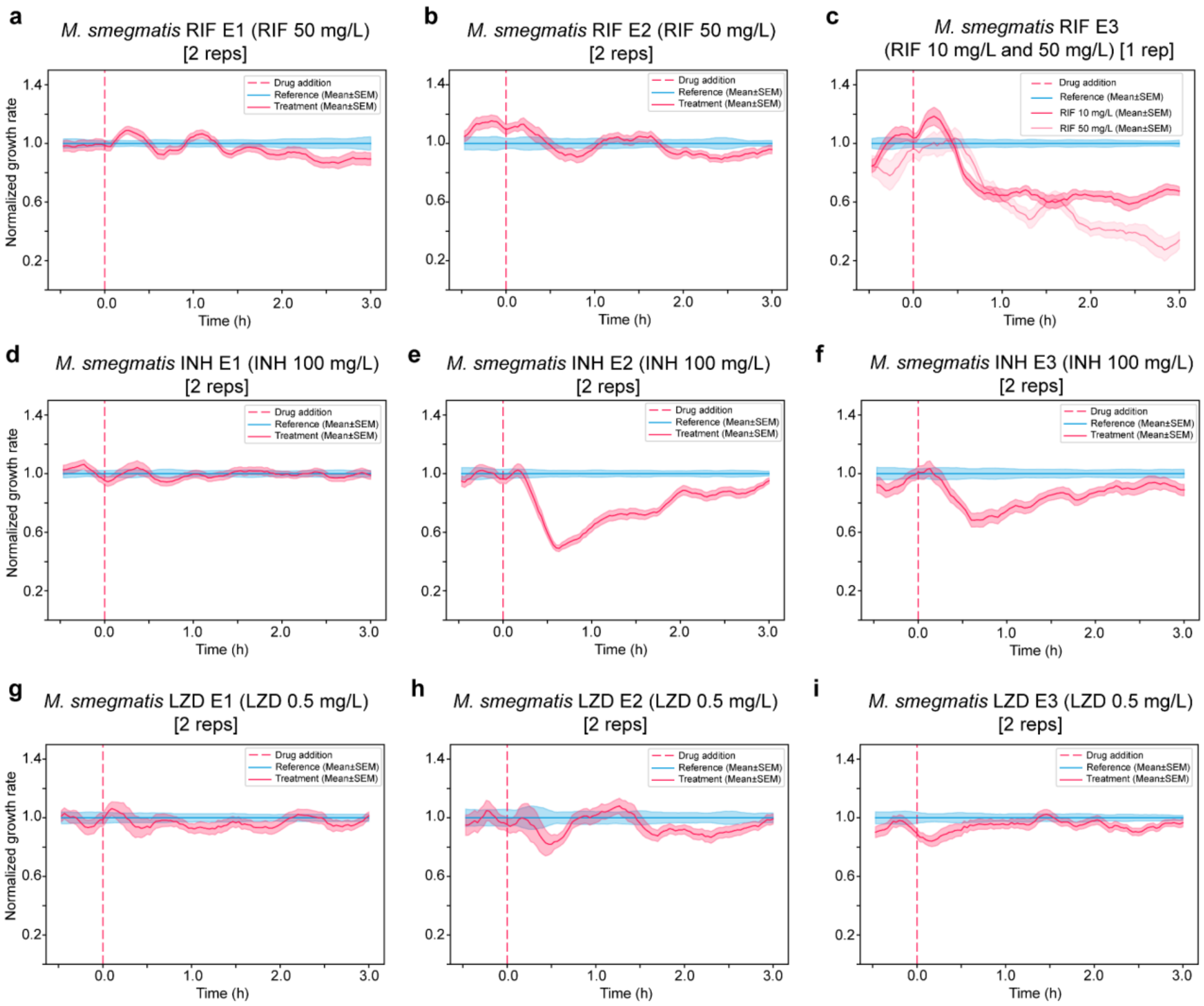

Supplementary Figure 8. Fast detection of resistant strains. pDST profiles of lab-evolved resistant strains derived from *M. smegmatis* NCTC 8159 including (**a**) *M. smegmatis* RIF E1, (**b**) *M. smegmatis* RIF E2, (**c**) *M. smegmatis* RIF E3 in rifampicin treatment; (**d**) *M. smegmatis* INH E1, (**e**) *M. smegmatis* INH E2, (**f**) *M. smegmatis* INH E3 in isoniazid treatment; and (**g**) *M. smegmatis* LZD E1, (**h**) *M. smegmatis* LZD E2, (**i**) *M. smegmatis* LZD E3 in linezolid treatment. Data were pooled from the number of biological replications indicated in each graph.

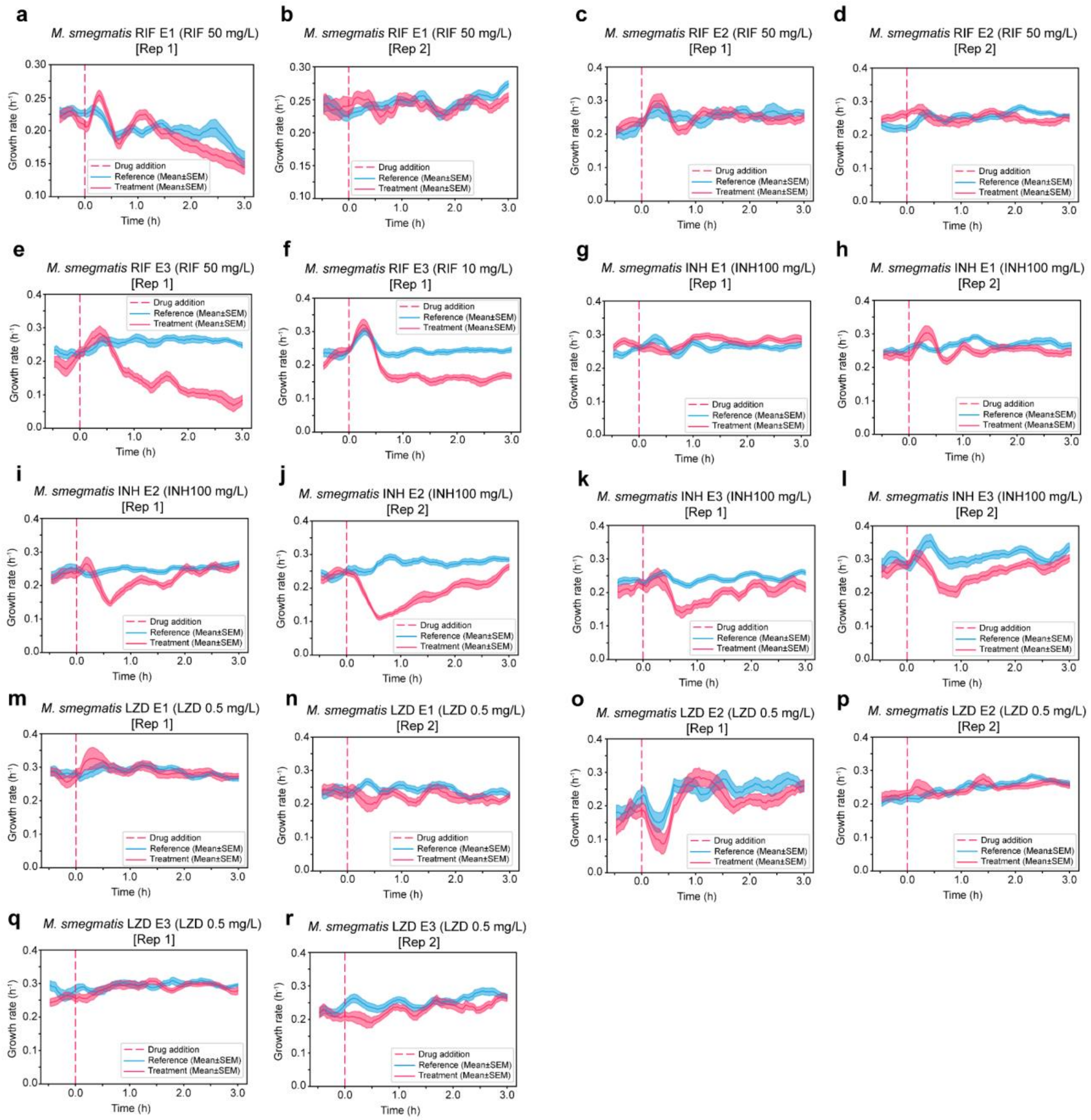

Supplementary Figure 9. The growth rates of resistant strains of *M. smegmatis* NCTC 8159 in response to drug treatments were measured across two biological replicates from different fast pDST experiments. **a - b**. *M. smegmatis* NCTC 8159 RIF E1 in rifampicin (RIF) 50 mg/L. **c - d**. *M. smegmatis* NCTC 8159 RIF E2 in rifampicin (RIF) 50 mg/L. **e - f**. *M. smegmatis* NCTC 8159 RIF E3 in rifampicin (RIF) 50 mg/L and 10 mg/L, respectively. **g - h**. *M. smegmatis* NCTC 8159 INH E1 in isoniazid (INH) 100 mg/L. **i - j**. *M. smegmatis* NCTC 8159 INH E2 in isoniazid (INH) 100 mg/L. **k - l**. *M. smegmatis* NCTC 8159 INH E3 in isoniazid (INH) 100 mg/L. **m - n**. *M. smegmatis* NCTC 8159 LZD E1 in linezolid (LZD) 0.5 mg/L. **o - p**. *M. smegmatis* NCTC 8159 LZD E2 in linezolid (LZD) 0.5 mg/L. **q - r**. *M. smegmatis* NCTC 8159 LZD E3 in linezolid (LZD) 0.5 mg/L.

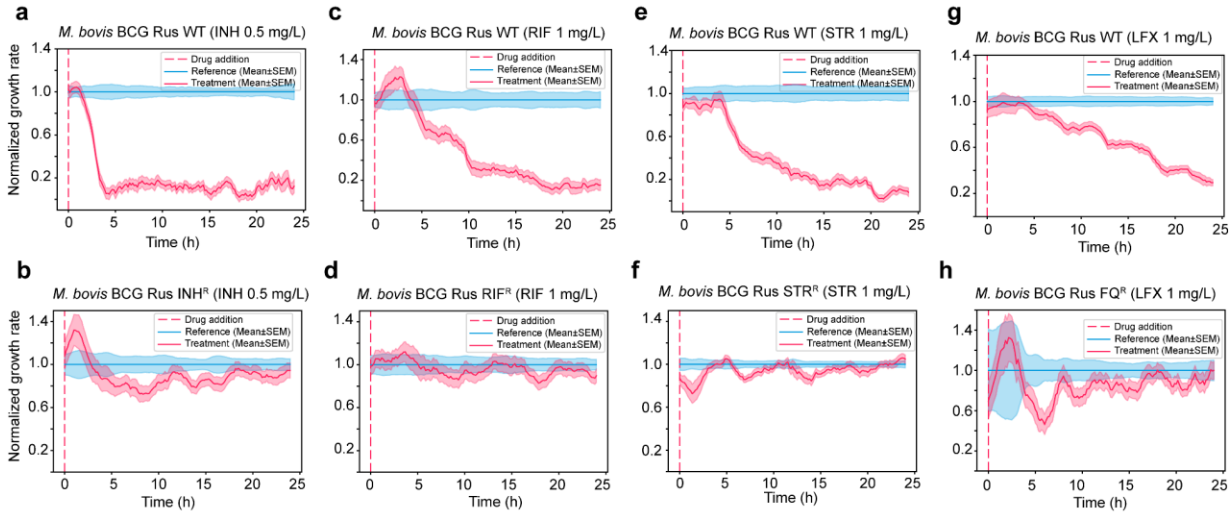

Supplementary Figure 10. pDST profiles of susceptible and resistant *M. bovis* BCG Russia at critical concentrations (CCs) from two biological replications. *M. bovis* BCG Russia WT (**a**) and *M. bovis* BCG Russia INH^{R} (**b**) in INH 0.5 mg/L; *M. bovis* BCG Russia WT (**c**) and *M. bovis* BCG Russia RIF^{R} (**d**) in RIF 1 mg/L; *M. bovis* BCG Russia WT (**e**) and *M. bovis* BCG Russia STR^{R} (**f**) in STR 1 mg/L; and *M. bovis* BCG Russia WT (**g**) and *M. bovis* BCG Russia FQ^{R} (**h**) in LFX 1 mg/L.

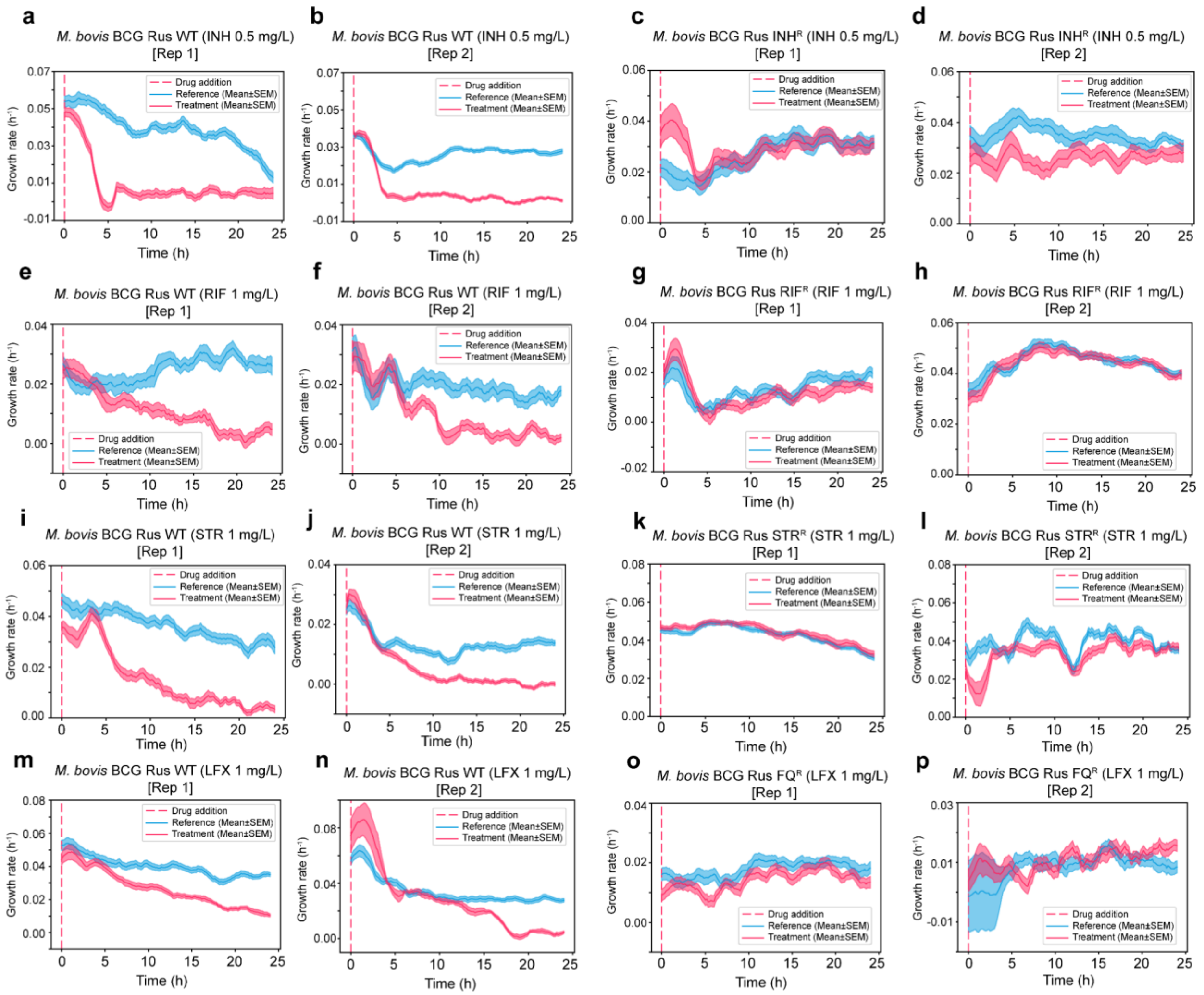

Supplementary Figure 11. The growth rates of susceptible and resistant *M. bovis* BCG Russia in response to drug treatments were measured across two biological replicates from different pDST experiments. *M. bovis* BCG Russia WT (**a** and **b**) and *M. bovis* BCG Russia INH^R (**c** and **d**) in INH 0.5 mg/L; *M. bovis* BCG Russia WT (**e** and **f**) and *M. bovis* BCG Russia RIF^R (**g** and **h**) in RIF 1 mg/L; *M. bovis* BCG Russia WT (**i** and **j**) and *M. bovis* BCG Russia STR^R (**k** and **l**) in STR 1 mg/L; and *M. bovis* BCG Russia WT (**m** and **n**) and *M. bovis* BCG Russia FQ^R (**o** and **p**) in LFX 1 mg/L.

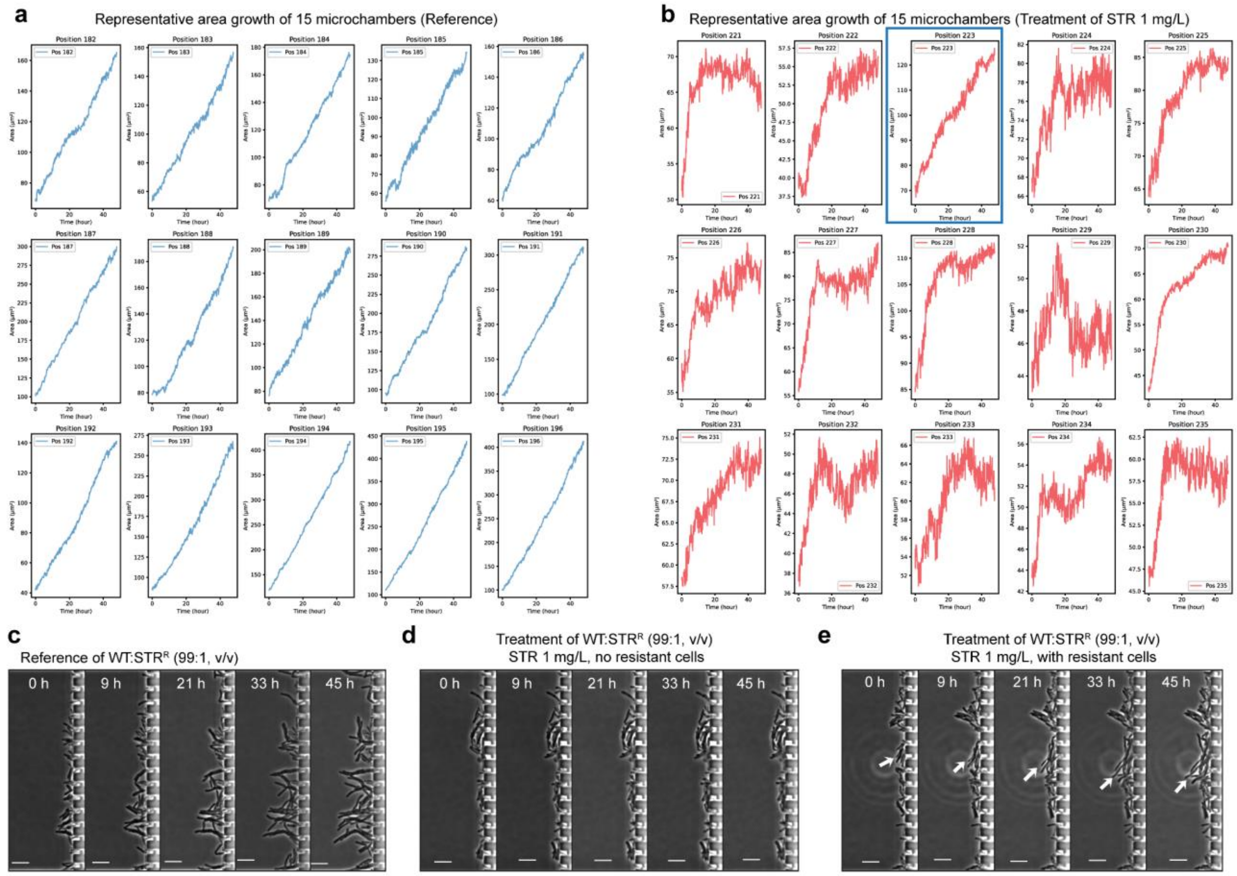

Supplementary Figure 12. Detection of heteroresistant infection (WT:STR^R) at the detection limit of 1%. Representative area growth of 15 individual microchambers of the reference **(a)** and the treatment in STR 1 mg/L **(b)**. The blue box in **(b)** shows the area growth curve of the position (microchamber) potentially containing resistant cells (panel **(e)**). Time-lapse representative images of the reference of WT:STR^R (99:1, v/v) (**c**), treatment of WT:STR^R (99:1, v/v) in STR 1 mg/L, no resistant cells (**d**), and treatment of WT:STR^R (99:1, v/v) in STR 1 mg/L, with resistant cells (**e**). The white arrows point to the resistant cells. Scale bars are 5 µm. We supplied supplementary video 4 for panel **(e)**. Supplementary video 5 shows a microchamber with resistant cells located separately from the susceptible population in the microchamber.

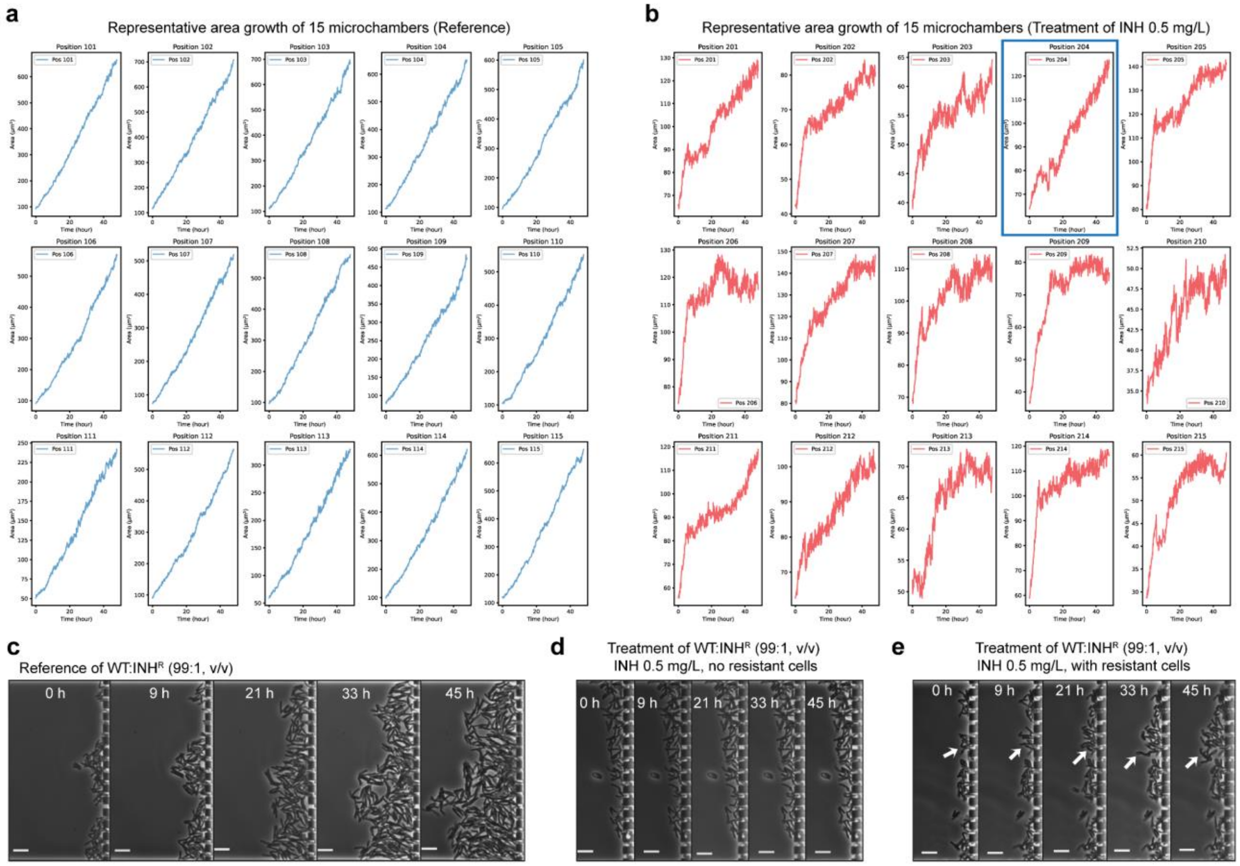

Supplementary Figure 13. Detection of heteroresistant infection (WT:INH^R) at the detection limit of 1%. Representative area growth of 15 individual microchambers of the reference **(a)** and the treatment in INH 0.5 mg/L **(b)**. The blue box in **(b)** shows the area growth curve of the position (microchamber) potentially containing resistant cells. Time-lapse representative images of the reference of WT:INH^R (99:1, v/v) (**c**), treatment of WT:INH^R (99:1, v/v) in INH 0.5 mg/L, no resistant cells (**d**), and treatment of WT:INH^R (99:1, v/v) in INH 0.5 mg/L, with resistant cells (**e**). The white arrows point to the resistant cells. Scale bars are 5 µm.

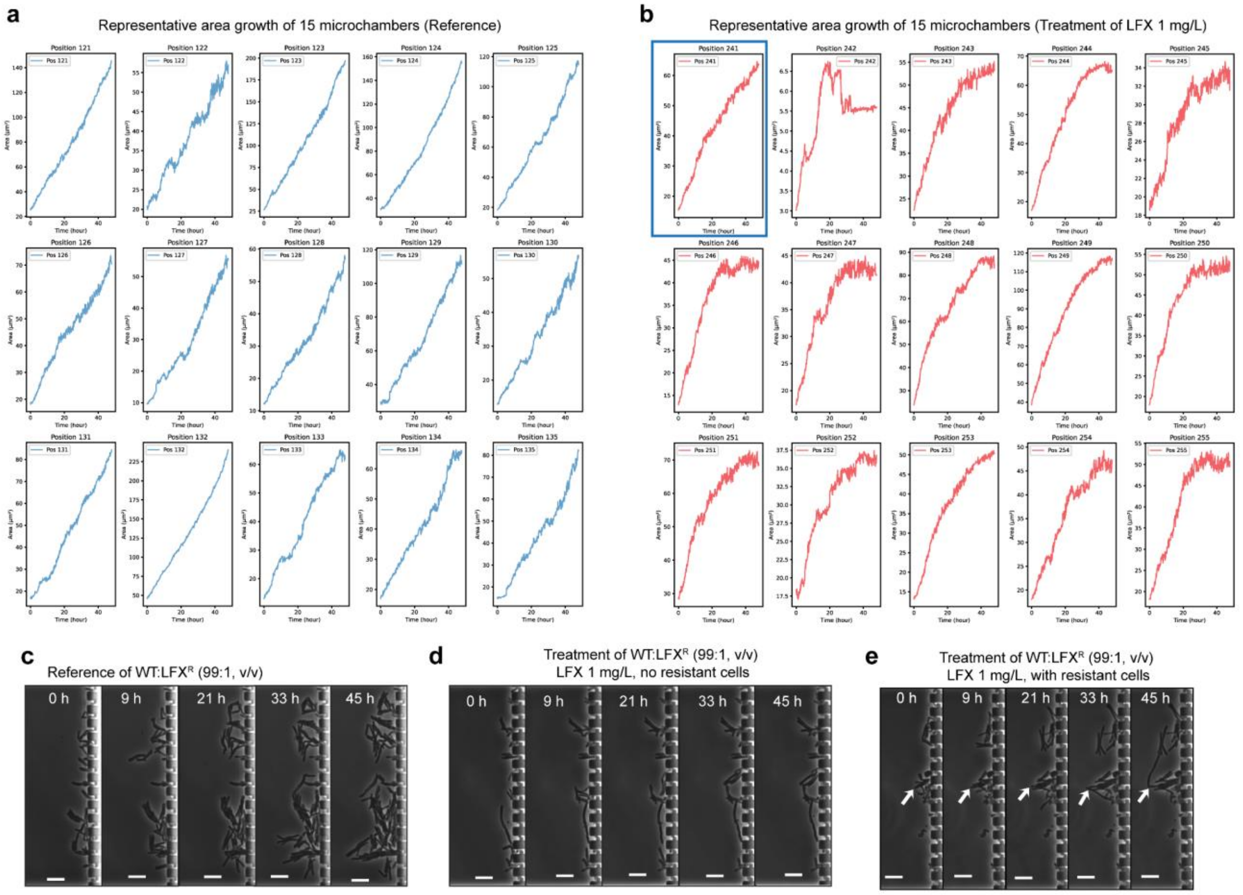

Supplementary Figure 14. Detection of heteroresistant infection (WT:FQ^R) at the detection limit of 1%. Representative area growth of 15 individual microchambers of the reference **(a)** and the treatment in LFX 1 mg/L **(b)**. The blue box in **(b)** shows the area growth curve of the position (microchamber) potentially containing resistant cells. Time-lapse representative images of the reference of WT:FQ^R (99:1, v/v) (**c**), treatment of WT:FQ^R (99:1, v/v) in LFX 1 mg/L, no resistant cells (**d**), and treatment of WT:FQ^R (99:1, v/v) in LFX 1 mg/L, with resistant cells (**e**). The white arrows point to the resistant cells. Scale bars are 5 µm.

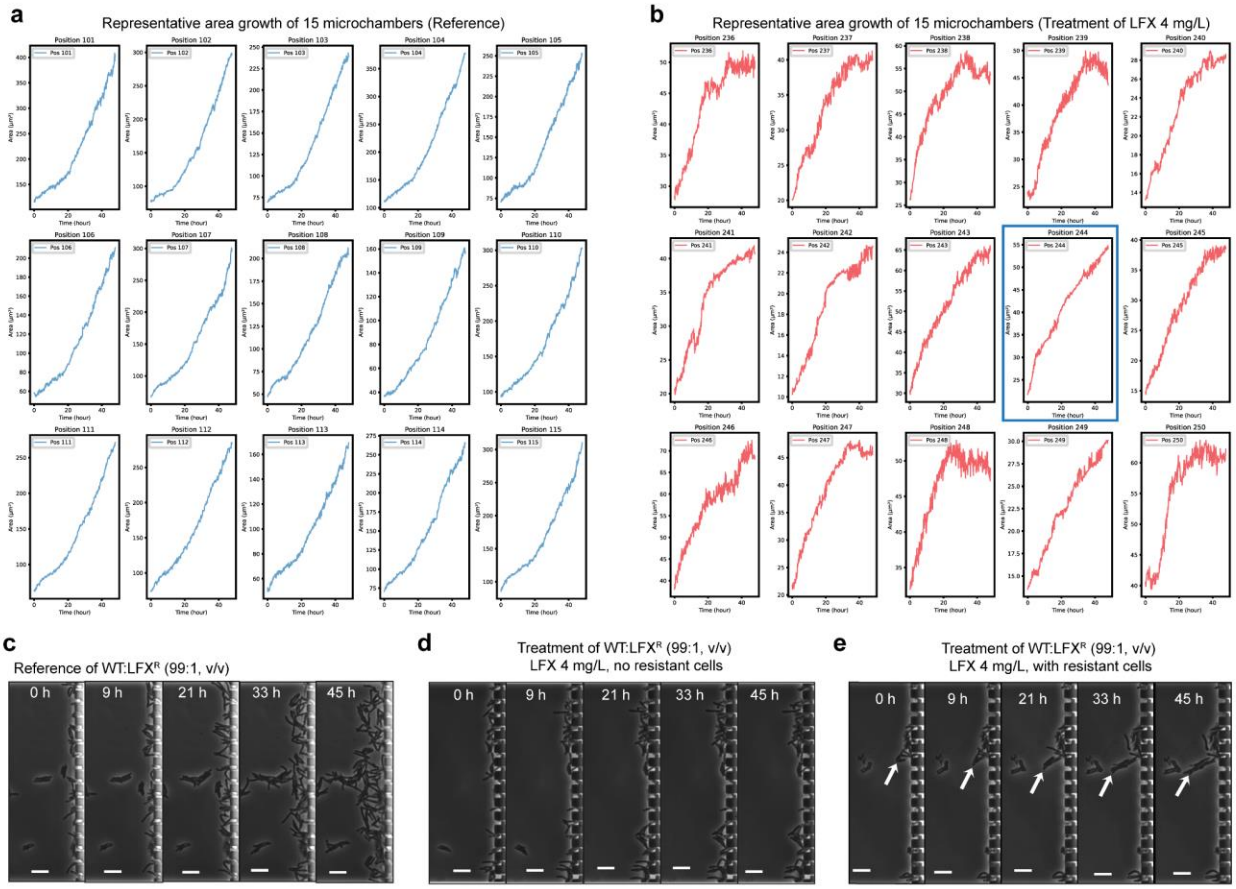

Supplementary Figure 15. Detection of heteroresistant infection (WT:FQ^R) at the detection limit of 1%. Representative area growth of 15 individual microchambers of the reference **(a)** and the treatment in LFX 4 mg/L **(b)**. The blue box in **(b)** shows the area growth curve of the position (microchamber) potentially containing resistant cells. Time-lapse representative images of the reference of WT:FQ^R (99:1, v/v) (**c**), treatment of WT:FQ^R (99:1, v/v) in LFX 4 mg/L, no resistant cells (**d**), and treatment of WT:FQ^R (99:1, v/v) in LFX 4 mg/L, with resistant cells (**e**). The white arrows point to the resistant cells. Scale bars are 5 µm.

Supplementary Table 1. Training/fine-tuning parameters of models for mycobacteria using Omnipose network [2]

No.	Model	Training parameters
1.	Omnipose	default Omnipose model for bacteria phase contrast images
2.	Mycobact_1	This model was trained from scratch with 4000 epochs, 2 classes, and a learning rate of 0.1; We used 240 images (120 phase images and 120 corresponding masks) of the training dataset.
3.	Mycobact_2	This model was trained from scratch with 4000 epochs, 3 classes, and a learning rate of 0.05; We used 240 images (120 phase images and 120 corresponding masks) of the training dataset.
4.	Mycobact_3	This model was trained from scratch with 4000 epochs, 3 classes, and a learning rate of 0.05; We used 738 images (369 phase images and 369 corresponding masks) of the training dataset. Of 369 phase images, 249 were from the Omipose training dataset, and 120 were from mycobacterial data.
5.	Mycobact_4	This model was trained from scratch with 8000 epochs, 3 classes, and a learning rate of 0.01; We used 738 images (369 phase images and 369 corresponding masks) of the training dataset. Of 369 phase images, 249 were from the Omipose training dataset, and 120 were from mycobacterial data.
6.	Mycobact_5	This model was fine-tuned from the default Omnipose model for bacteria phase contrast images with 1300 epochs, 3 classes, and a learning rate of 0.1; We used 240 images (120 phase images and 120 corresponding masks) of the training dataset.
7.	Mycobact_6	This model was fine-tuned from the default Omnipose model for bacteria phase contrast images with 4000 epochs, 3 classes, and a learning rate of 0.1; We used 240 images (120 phase images and 120 corresponding masks) of the training dataset.

Supplementary Table 2. MIC values of the tested strains were determined using the resazurin microtiter assay (REMA)[3] and EUCAST broth microdilution assay (BMDA)[4] (unit: mg/L)

No.	Strain	MIC					Remarks
		RIF	INH	EMB	LZD	BDQ	
1.	*M. bovis* BCG WT	0.06	0.5	2	0.5	0.125	ATCC - 35734
2.	*M. smegmatis* mc[2] 155 WT	10	2.5	1.6	0.16		From Leif Kirsebom
3.	*M. smegmatis* NCTC 8159 WT	10	50	1.6	0.5	0.05	[5]
4.	*M. smegmatis* NCTC 8159 RIF E1	>100 (50*)					
5.	*M. smegmatis* NCTC 8159 RIF E2	>100 (50)					
6.	*M. smegmatis* NCTC 8159 RIF E3	50 (50)					
7.	*M. smegmatis* NCTC 8159 INH E1		>100 (100)				
8.	*M. smegmatis* NCTC 8159 INH E2		>100 (100)				
9.	*M. smegmatis* NCTC 8159 INH E3		>100 (100)				
10.	*M. smegmatis* NCTC 8159 LZD E1				50 (0.5)		
11.	*M. smegmatis* NCTC 8159 LZD E2				12.5 (0.5)		
12.	*M. smegmatis* NCTC 8159 LZD E3				50 (0.5)		

(*) Values in the parentheses are the lab-evolved selective concentrations[5]

References

1. Baltekin, Ö., Boucharin, A., Tano, E., Andersson, D. I. & Elf, J. Antibiotic susceptibility testing in less than 30 min using direct single-cell imaging. *Proc. Natl. Acad. Sci. U. S. A.* **114**, 9170–9175 (2017).
2. Cutler, K. J. *et al.* Omnipose: a high-precision morphology-independent solution for bacterial cell segmentation. *Nat. Methods* **19**, 1438–1448 (2022).
3. Palomino, J.-C. *et al.* Resazurin microtiter assay plate: simple and inexpensive method for detection of drug resistance in Mycobacterium tuberculosis. *Antimicrob. Agents Chemother.* **46**, 2720–2722 (2002).
4. Schön, T. *et al.* Antimicrobial susceptibility testing of Mycobacterium tuberculosis complex isolates - the EUCAST broth microdilution reference method for MIC determination. *Clin. Microbiol. Infect.* **26**, 1488–1492 (2020).
5. Maeda, T., Kawada, M., Sakata, N., Kotani, H. & Furusawa, C. Laboratory evolution of Mycobacterium on agar plates for analysis of resistance acquisition and drug sensitivity profiles. *Sci. Rep.* **11**, 15136 (2021).
