# Supplementary material for: Phenotypic drug susceptibility testing for Mycobacterium tuberculosis variant bovis BCG in 12 hours
Source: Nat Commun. 2025 May 10;16:4366. doi: 10.1038/s41467-025-59736-9 (PMC12065818; doi:10.1038/s41467-025-59736-9)
Supplement: Supplementary file 2 — Description of Additional Supplementary Files [file 41467_2025_59736_MOESM2_ESM.pdf]

Description of Additional Supplementary Files

Supplementary video legends

File Name: Supplementary Video 1
Description: Deep neural network image-segmentation performance of phase contrast images from *M. bovis* BCG, related to Figure 2. **a.** Phase contrast images of *M. bovis* BCG WT with no antibiotic (reference). **b.** Phase contrast images of *M. bovis* BCG WT treated with INH 0.5 mg/L (treatment). **c**. Segmented mask of images in (a). **d**. Segmented mask of images in (b).

File Name: Supplementary Video 2
Description: Deep neural network image-segmentation performance of phase contrast images from *M. smegmatis* NCTC 8159, related to Figure 2. **a**. Phase contrast images of *M. smegmatis* NCTC 8159 WT with no antibiotic (reference). **b**. Phase contrast images of *M. smegmatis* NCTC 8159 WT treated with RIF 50 mg/L (treatment). **c**. Segmented mask of images in (a). **d**. Segmented mask of images in (b).

File Name: Supplementary Video 3
Description: Inhibition of *M. smegmatis* NCTC 8159 WT by Bedaquiline treatment (BDQ 2.5 mg/L) using FEP tubing, related to Supplementary Figure 7e. BDQ was added after 1 hour.

File Name: Supplementary Video 4
Description: Heteroresistance - phenotypic drug susceptibility test (pDST) of *M. bovis* BCG Russia WT and RpsL K43R (STR^R) (99:1, v/v) at 1 mg/L STR, related to Supplementary Fig. 12e and 12f. STR was added after 3 hours. Resistant cells are indicated by the white arrow.

File Name: Supplementary Video 5
Description: Heteroresistance - phenotypic drug susceptibility test (pDST) of *M. bovis* BCG Russia WT and RpsL K43R (STR^R) (99:1, v/v) at 1 mg/L STR. STR was added after 3 hours. Resistant cells are indicated by the white arrow. In this video, the resistant cells are separated from the susceptible population.
