# Supplementary material for: Phenotypic drug susceptibility testing for Mycobacterium tuberculosis variant bovis BCG in 12 hours
Source: Nat Commun. 2025 May 10;16:4366. doi: 10.1038/s41467-025-59736-9 (PMC12065818; doi:10.1038/s41467-025-59736-9)
Supplement: Supplementary file 8 — Reporting Summary [file 41467_2025_59736_MOESM8_ESM.pdf]

nature portfolio

Corresponding author(s): Johan Elf

Last updated by author(s): Apr 10, 2025

Reporting Summary

Nature Portfolio wishes to improve the reproducibility of the work that we publish. This form provides structure for consistency and transparency in reporting. For further information on Nature Portfolio policies, see our Editorial Policies and the Editorial Policy Checklist.

Statistics

For all statistical analyses, confirm that the following items are present in the figure legend, table legend, main text, or Methods section.

n/a	Confirmed	
☐	☒	The exact sample size (*n*) for each experimental group/condition, given as a discrete number and unit of measurement
☐	☒	A statement on whether measurements were taken from distinct samples or whether the same sample was measured repeatedly
☒	☐	The statistical test(s) used AND whether they are one- or two-sided<br>*Only common tests should be described solely by name; describe more complex techniques in the Methods section.*
☒	☐	A description of all covariates tested
☒	☐	A description of any assumptions or corrections, such as tests of normality and adjustment for multiple comparisons
☐	☒	A full description of the statistical parameters including central tendency (e.g. means) or other basic estimates (e.g. regression coefficient) AND variation (e.g. standard deviation) or associated estimates of uncertainty (e.g. confidence intervals)
☒	☐	For null hypothesis testing, the test statistic (e.g. *F*, *t*, *r*) with confidence intervals, effect sizes, degrees of freedom and *P* value noted<br>*Give P values as exact values whenever suitable.*
☒	☐	For Bayesian analysis, information on the choice of priors and Markov chain Monte Carlo settings
☒	☐	For hierarchical and complex designs, identification of the appropriate level for tests and full reporting of outcomes
☒	☐	Estimates of effect sizes (e.g. Cohen's *d*, Pearson's *r*), indicating how they were calculated

Our web collection on statistics for biologists contains articles on many of the points above.

Software and code

Policy information about availability of computer code

Data collection	Micro-Manager-1.4.23-20190429 was used to control microscope for image acquisition. Image preprocessing was made using custom MATLAB (2022a) code. Cell segmentation was performed using Omnipose.
Data analysis	Custom MATLAB code was used for image preprocessing. Analysis of preprocessed data were done using Jupyter lab version 2.3.2 and Python version 3.8.5 in NVIDIA PyTorch Container version 22.03. The analysis code can be found at (https://doi.org/10.17044/scilifelab.26927146).

For manuscripts utilizing custom algorithms or software that are central to the research but not yet described in published literature, software must be made available to editors and reviewers. We strongly encourage code deposition in a community repository (e.g. GitHub). See the Nature Portfolio guidelines for submitting code & software for further information.

Data

Policy information about availability of data

All manuscripts must include a data availability statement. This statement should provide the following information, where applicable:

- Accession codes, unique identifiers, or web links for publicly available datasets
- A description of any restrictions on data availability
- For clinical datasets or third party data, please ensure that the statement adheres to our policy

All experimental data generated in this study have been deposited in the SciLifeLab Data Repository at https://doi.org/10.17044/scilifelab.2692714671. Source data are provided with this paper.

Research involving human participants, their data, or biological material

Policy information about studies with human participants or human data. See also policy information about sex, gender (identity/presentation), and sexual orientation and race, ethnicity and racism.

Reporting on sex and gender	We do not include human participants in our work.
Reporting on race, ethnicity, or other socially relevant groupings	We do not include human participants in our work.
Population characteristics	We do not include human participants in our work.
Recruitment	We do not include human participants in our work.
Ethics oversight	We do not include human participants in our work.

Note that full information on the approval of the study protocol must also be provided in the manuscript.

Field-specific reporting

Please select the one below that is the best fit for your research. If you are not sure, read the appropriate sections before making your selection.

☒ Life sciences ☐ Behavioural & social sciences ☐ Ecological, evolutionary & environmental sciences

For a reference copy of the document with all sections, see nature.com/documents/nr-reporting-summary-flat.pdf

Life sciences study design

All studies must disclose on these points even when the disclosure is negative.

Sample size	No required sample size was predetermined in advance as we are not testing a hypothesis.
Data exclusions	Images with empty or extremely out-of-focus positions or bad segmentation were discarded in further analysis.
Replication	The number of replications was indicated in each figure legend. Multiple biological replication data were shown in Supplementary Figures.
Randomization	Randomization was not necessary as we are not testing a hypothesis.
Blinding	Blinding was not necessary as we are not testing a hypothesis.

Reporting for specific materials, systems and methods

We require information from authors about some types of materials, experimental systems and methods used in many studies. Here, indicate whether each material, system or method listed is relevant to your study. If you are not sure if a list item applies to your research, read the appropriate section before selecting a response.

Materials & experimental systems

n/a	Involved in the study	
☒	☐	Antibodies
☒	☐	Eukaryotic cell lines
☒	☐	Palaeontology and archaeology
☒	☐	Animals and other organisms
☒	☐	Clinical data
☒	☐	Dual use research of concern
☒	☐	Plants

Methods

n/a	Involved in the study	
☒	☐	ChIP-seq
☒	☐	Flow cytometry
☒	☐	MRI-based neuroimaging

Plants

Seed stocks	We do not include any plants in our work.
Novel plant genotypes	We do not include any plants in our work.
Authentication	We do not include any plants in our work.
